# Reply to: Fluid signal suppression characteristics of 3D-FLAIR with a T2 selective inversion pulse in the skull base

**DOI:** 10.1038/s41467-023-40509-1

**Published:** 2023-08-16

**Authors:** Mehmet Sait Albayram, Garrett Smith, Onder Albayram

**Affiliations:** 1 Department of Radiology, University of Florida College of Medicine, Gainesville, FL 32610 USA; 2 Department of Pathology and Laboratory Medicine, Medical University of South Carolina, Charleston, SC 29425 USA; 3 Department of Neuroscience, Medical University of South Carolina, Charleston, SC 29425 USA

**Keywords:** Neurophysiology, Imaging

**replying to** S. Naganawa et al. *Nature Communications* 10.1038/s41467-023-40507-3 (2023)

First, we would like to thank Dr. Naganawa and his colleagues for their interest in our article. We are happy to address their concerns regarding our article^1^. Their main concern is that the signals we detected in the ventral region represent artifacts rather than lymphatic signals^2^. In the first revision of our manuscript, we detailed the effects of TR, TE, and TI variation in our MR technique on signals measured in a healthy volunteer. Unfortunately, we removed these data from the manuscript in later drafts after including the results of our phantom study. Our study used a 3D-FLAIR technique with a T2-selective inversion pulse. For comparison, Dr. Naganawa presented four different 3D-FLAIR techniques: T2-selective inversion pulse, T2-preparation pulses, conventional inversion pulse similar to our techniques, and conventional inversion using longer repetition time of 8000 (TR/TE/TI: 8000/387/2370)^2^. We will only discuss the first 3, as the fourth technique uses parameters significantly different from those we used (TR/TE/TI: 5000/387/1800).

The study by Naganawa et al.^2^ reported that signals in the ventral region (white arrows) were similarly visible in images obtained with the T2-selective IR (Naganawa et al. Fig. 2A, C), and not in images obtained from their volunteer case with the conventional inversion pulse (Naganawa et al. Figs. 1 and 2 (Fig. 1B, D, F). They concluded that the signals seen in the ventral areas might be artifacts rather than protein-rich lymphatic fluid signals due to the absence of the ventral signal in conventional IR. We agree that the ventral signals cannot be seen in conventional IR series in Naganawa et al. Figs. 1 and 2; however, it is also difficult to distinguish the gray matter, middle cerebellar peduncle, and cerebellar cortex well in conventional IR series (Fig. 1B, D, F). The entire parenchyma, including gray matter, white matter, and middle cerebellar peduncles, are similar in signal intensity with the conventional IR technique (white boxes and blue arrows), whereas these structures can be seen clearly with T2-selective IR. We also see a significant dorsal lymphatic signal decrease (white arrowheads) in conventional IR images (Fig. 1F) compared to T2-selective IR images (Fig. 1E) in Naganawa et al. Figure 1. Conventional IR cannot differentiate tissue-fluid differences well and may, therefore, also fail to detect protein-solute-rich lymphatic fluid. As a result, we do not believe that the absence of signal in conventional IR alone suggests the absence of lymphatic signal.

**Fig. 1:**
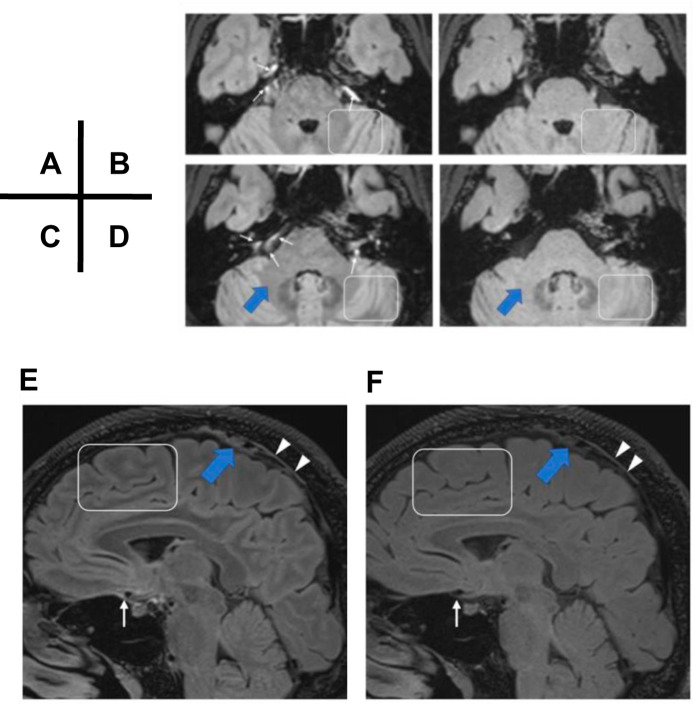
Ventral and dorsal lymphatic/ISF signal differences between the T2-selective IR and conventional IR. Lymphatic fluid/ISF signals in the ventral and dorsal regions are similarly visible in images obtained with T2-selective IR (**A**, **C**, **E**), but not in images obtained with the conventional inversion pulse in the study’s volunteer case (**B**, **D**, **F**). The ventral signal (white arrows) and dorsal signal (white arrowheads) cannot be seen in conventional IR series. The entire parenchyma, including gray matter, white matter, and middle cerebellar peduncles, present with similar signal intensity with the conventional IR technique (**B**, **D**, **F**), whereas these structures can be seen clearly with T2-selective IR sequences (**A**, **C**, **E**) (white boxes and blue arrows). (Adapted from Figs. 1 and 2 of Naganawa et al., *Nat Commun*. submitted)^2^.

We can also test our hypotheses regarding lymphatic Cerebrospinal Fluid (CSF) - Interstitial Fluid (ISF) visualization in other protein-rich regions in the calvarium outside of the brain. There is a 7.2-fold greater protein concentration in perilymphatic space (PLS) versus endolymphatic area (ELS) in the guinea pig^3^, which the current conventional 3D-FLAIR sequence cannot capture without contrast. On December 20, 2021, Fukutomi et al. published “Visualization of the saccule and utricle with non-contrast-enhanced FLAIR sequences”^4^. This study described how conventional 3D FLAIR IR imaging techniques could not differentiate chemical differences between PLS and ELS. When using T2 prep inversion pulse sequences, however, the PLS and ELS could be discerned. They concluded that a specific combination of T2 Prep time and TI from high-resolution 3D-FLAIR could differentiate ELS from PLS and even delineate the saccule from the utricle without gadolinium injection^4^. Therefore, with these findings, we believe that 3D FLAIR with a T2-selective inversion pulse allows for evaluating solute-protein-rich fluid, whereas 3D conventional IR imaging techniques do not. We also found that PLS and ELS can be differentiated on 3D FLAIR technique without contrast usage during our data analyses.

Recently Jacob et al.^5^ used the T1 SPACE DANTE MR sequence to accurately segregate the slow-flow circuits of the lymphatic vessels from the faster-flow circuits of arteries, veins, venules, and CSF. The slower-flow lymphatic circuit was concentrated in the perisinusoidal areas along the superior sagittal, straight, transverse, sigmoid, and cavernous sinus, standing apart from venous sinuses and veins and included vessel-like compartments associated with flattened vesicles. In the neck region, extracranial lymphatics connected dural lymphatic vessels with cervical lymph nodes. The exit of lymphatics from the skull was observed along blood vessels through the jugular foramen and along the carotid canal, the superior orbital fissure, the foramen rotundum, and the foramen ovale for lymphatics of the cavernous sinus. These findings corroborate our hypothesized dorsal lymphatic flow and structures and further suggest significant ventral lymphatic flow at the skull base in humans at the level of jugular foremen, sellar region, and 5^th^ nerve pathway. Although we detected a strong signal in the anterior cranial fossa around the olfactory nerves at the level of the internal auditory canal, Jacob et al. did not detect any lymphatic signal in these regions. These differences might relate to internal marker variances (gadolinium vs. solid-rich interstitial fluid). However, we cannot completely rule out possible artifacts in these regions. Therefore, we agree that prospective and well-designed live-human studies supported by histopathology are needed for clarification. Jacob et al. did not specifically report on the internal auditory canal region. Therefore, again, the dural FLAIR signal at the level of the internal auditory canal is likely a lymphatic signal similar to other dural surfaces. Our signals that extend beyond the dura into subdural or subarachnoid spaces may represent artifacts or CSF-ISF exchange in this region toward the dural lymphatics system from subdural-subarachnoid spaces. At this time, there are not ample data regarding CSF-ISF exchange at the level of the internal auditory canal in the subdural-subarachnoid spaces. Recently, subarachnoid lymphatic structures have been shown by Mezey et al.^6^, but more investigation is needed to confirm this finding. The possibility of artifact at the level of the internal auditory canal in the subdural and subarachnoid spaces could not be excluded. Further histopathological studies will be needed.

We have also created the step-by-step protocols used in the original publication of *Protocol Exchange*^7^.

We want to thank Dr. Naganawa and his colleagues again for their interest in our article. We hope the responses above address their concerns.

## Reporting summary

Further information on research design is available in the Nature Portfolio Reporting Summary linked to this article.

### Supplementary information

Reporting Summary
